# Obituary

**Published:** 1877-02

**Authors:** 


### Obituary.

In Chicago, January 7, 1877, died, ORRIN COOLEY, M.D, aged 33 years.

Dr. Cooley was a native of Massachusetts. He took his degree at the University of Pennsylvania, in 1869. For a year after his graduation he was house surgeon at the State Asylum for Inebriates of Pennsylvania. He settled in Chicago a few months before the great fire, by which he sustained severe loss.

He was well but not widely known in the profession. Those who knew him esteemed him. He was an honorable physician, a student, a scholar, a truly modest gentleman — altogether a man the profession could illy afford to lose.

He had delivered in the Chicago Medical College, some years ago, several courses of lectures on surgical anatomy, which were distinguished for their clearness and excellence.

